# Candidemia in the critically ill: initial therapy and outcome in mechanically ventilated patients

**DOI:** 10.1186/1471-2253-13-37

**Published:** 2013-10-30

**Authors:** Marcela A Ferrada, Andrew A Quartin, Daniel H Kett, Michele I Morris

**Affiliations:** 1 Department of Internal Medicine, 1120 N.W. 14th Avenue, (R-21), Clinical Research Building, Room 842, Miami, FL 33136, USA; 2 Division of Pulmonary, Critical Care, and Sleep Medicine, Miami, FL 33136, USA; 3 Division of Infectious Diseases, University of Miami Miller School of Medicine, 1120 N.W. 14th Avenue, (R-21), Clinical Research Building, Room 842, Miami, FL 33136, USA; 4 Critical Care Medicine (111), Miami VA Hospital, 1201 N.W. 16th St, Miami, FL 33125, USA

**Keywords:** Candidemia, Mechanical ventilation, Intensive care, Azoles, Echinocandins

## Abstract

**Background:**

Mortality among critically ill patients with candidemia is very high. We sought to determine whether the choice of initial antifungal therapy is associated with survival among these patients, using need for mechanical ventilatory support as a marker of critical illness.

**Methods:**

Cohort analysis of outcomes among mechanically ventilated patients with candidemia from the 24 North American academic medical centers contributing to the Prospective Antifungal Therapy (PATH) Alliance registry. Patients were included if they received either fluconazole or an echinocandin as initial monotherapy.

**Results:**

Of 5272 patients in the PATH registry at the time of data abstraction, 1014 were ventilated and concomitantly had candidemia, with 689 eligible for analysis. 28-day survival was higher among the 374 patients treated initially with fluconazole than among the 315 treated with an echinocandin (66% versus 51%, P < .001). Initial fluconazole therapy remained associated with improved survival after adjusting for non-treatment factors in the overall population (hazard ratio .75, 95% CI .59–.96), and also among patients with *albicans* infection (hazard ratio .62, 95% CI .44–.88). While not statistically significant, fluconazole appeared to be associated with higher mortality among patients infected with *glabrata* (HR 1.13, 95% CI .70–1.84).

**Conclusions:**

Among ventilated patients with candidemia, those receiving fluconazole as initial monotherapy were significantly more likely to survive than those treated with an echinocandin. This difference persisted after adjustment for non-treatment factors.

## Background

Candidemia is a major cause of nosocomial bloodstream infection in the United States [1,2], particularly among intensive care unit (ICU) patients [3,4]. Increasing attributable mortality from candidemia has prompted research into the role of early diagnosis and treatment in improving outcomes [3,5,6].

Rapid initiation of appropriate antibiotic therapy has resulted in better survival among patients with septic shock and ventilator-associated pneumonia [7-9]. Similarly, improved outcomes have been reported with early treatment of suspected fungal infections, though often patients are started on therapy before speciation [10-12]. Thus, the epidemiology of candidemia may impact the choice of initial therapy. Non-*albicans* species comprise approximately half of *Candida* bloodstream infections, with increasing isolation of species less susceptible to fluconazole [13].

The introduction of the echinocandins has expanded the options available for the treatment of *Candida* infections in the critically ill. Whether these newer medications are preferable to fluconazole as initial antifungal therapy in the ICU remains unresolved. While recent guidelines recommend the use of echinocandins for moderately severe to severely ill patients with candidemia [14], previous comparisons of azoles and echinocandins have included only limited populations of critically ill patients [15-17]. Perhaps more importantly, controlled trials have not evaluated initial therapy; enrollment criteria permitted up to 48 hours of antifungal therapy before initiation of study drug [16,18,19]. Recent critiques of the guideline development process suggest the need for studies that address actual clinical circumstance [20,21].

A large controlled trial of initial antifungal therapy for critically ill patients with candidemia promises to be extremely challenging logistically. The need for therapy is often unrecognized until *Candida* is identified in culture. Once fungemia is recognized, evidence suggests therapy should be started as quickly as possible, leaving little time for trial related activities such as consent and randomization. Absent the more definitive data from such a trial, a cohort study of critically ill patients treated with either fluconazole or an echinocandin as initial therapy should inform current practice and point the way forward for further investigations. We therefore analyzed the epidemiology and outcomes of patients included in a large, multicenter mycosis registry who required mechanical ventilatory support, had candidemia, and received initial monotherapy of fluconazole or an echinocandin.

## Methods

### Data collection

Data were extracted from the Prospective Antifungal Therapy (PATH) Alliance registry, created in 2004 to collect information about patients with invasive fungal infections at 24 centers in the United States and Canada [22]. Qualifying patients entered in the PATH registry through September 17, 2008 were followed for survival through 28 days. The Human Subject Research Office of the University of Miami Miller School of Medicine reviewed and approved the protocol (HSRO 20060202).We submitted a proposal to the registry sponsors to review all candidemia cases entered in the registry that were reported in mechanically ventilated patients, and we were granted permission to analyze this subgroup of the PATH database.

Patients were considered critically ill if mechanically ventilated at the time the initial positive culture was obtained. Critically ill patients were included in the analysis if they were diagnosed with candidemia and received initial antifungal monotherapy with either an echinocandin or fluconazole from zero to six days after the index culture was obtained. Patients were excluded if they had a previous or concurrent fungal infection recorded in the PATH registry.

Data regarding delay between index culture and initiation of therapy, whether therapy was started before cultures were known to be positive (defined as empiric therapy), antifungal agents used, *Candida* species found on the index culture, and antifungal agents received in the month prior to the index culture were recorded. The PATH database does not fully distinguish prior non-directed antifungal exposure from empiric therapy, counting antifungal courses initiated before identification of candidemia and continued to confirmation of infection as both directed therapy and prior exposure.

Possible risk factors for candidemia or death were recorded including: malignancies; diabetes mellitus; corticosteroid use, or receipt of other immunosuppressive medications or total body irradiation in the preceding month, or either (any immunosuppressive therapy); neutropenia, (fewer than 500 neutrophils/mm^3^) within the preceding month; post-operative status; solid organ transplant recipient; HIV infection; serum creatinine > 2 mg/dL, receiving renal replacement therapy, or either (any renal dysfunction); and transaminitis (alanine aminotransferase or aspartate aminotransferase > 100 U/L), total bilirubin > 3 mg/dL, or either (any liver disease). Patients were followed for up to 12 weeks following the initial positive culture.

### Statistical analysis

Population characteristics were compared across initial therapy using Fisher’s exact test, the χ^2^ test, and t tests or Wilcoxon’s test, as appropriate for data type and distribution. The principal outcome, survival through 28 days within initial antifungal therapy groups, was evaluated using the log-rank test. Day zero was the first day antifungal therapy was administered. P < .05 was considered a significant difference between treatment groups.

Propensity for use of echinocandins as initial therapy was modeled using logistic regression. Candidate predictors included all demographic and risk factors, time delay between obtaining the index culture and starting antifungal therapy, and whether therapy was started empirically. *Candida* species was not considered, as it would not typically be known when candidemia is first recognized and treatment prescribed. Variables were chosen using forward stepwise selection, requiring P < .20 to enter or remain in the model. The discrimination capacity of the propensity model was tested using the C statistic.

The relationships of individual factors with initial antifungal therapy were assessed using proportional hazards models. The hazard ratio associated with fluconazole as initial therapy was calculated in the subpopulations with and without each risk factor in univariate models, and the P value for inclusion of the interaction term between each risk factor and initial therapy calculated from the corresponding bivariate models for the entire population.

A treatment independent risk term (TIRT) was calculated for each patient. A proportional hazards model of survival through 28 days was created using as candidate predictors all demographic and risk factors, time delay between index culture and antifungal therapy initiation, whether therapy was started empirically, and propensity for echinocandin use. Variables were chosen for inclusion using forward stepwise selection with switching, requiring P < .20 to enter or remain in the model. The TIRT was calculated for each patient from this model (the coefficient for TIRT being unity in a univariate model).

The adjusted hazard for death with initial treatment of fluconazole across the entire population was calculated by including initial therapy and TIRT in a bivariate proportional hazards model. Because the PATH registry does not clearly distinguish prior from empiric antifungal therapy, this analysis was repeated for subpopulations defined by receipt of prior or empiric therapy to ensure robustness of results. Similar analyses were carried out for the subpopulations infected with each *Candida* species after determining that TIRT appropriately described treatment independent risk for each subpopulation.

## Results and discussion

### Population characteristics

**Figure 1:**
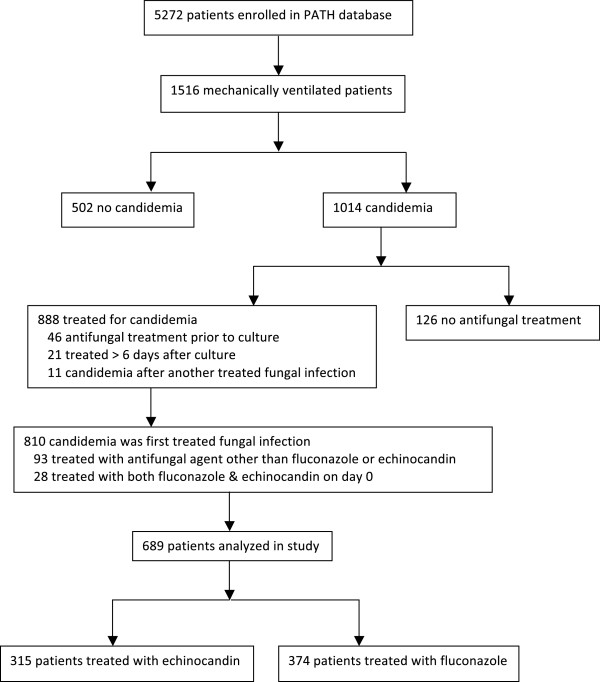
PATH study patient distribution and study enrollment for analysis.

The PATH registry included 1516 mechanically ventilated patients. Of these, 810 had candidemia as their first PATH registered infection and received initial therapy within zero to six days of the index blood culture. An antifungal other than an echinocandin or fluconazole was initially administered to 93 patients, and another 28 received both an echinocandin and fluconazole on day zero. Thus, 689 patients qualified for this analysis, 315 (46%) treated initially with an echinocandin and 374 (54%) with fluconazole (Figure 1).

Patients given an echinocandin as initial therapy were more likely to have received immunosuppressive therapies in the preceding month and to have renal dysfunction at the time of diagnosis than patients treated with fluconazole (P < .001 for each) (Table 1). Hematologic malignancies were rare but more common among patients treated with echinocandins [17 (5%) echinocandin patients and 8 (2%) fluconazole patients], as was recent neutropenia [20 (6%) echinocandin patients and 2 (1%) fluconazole patients]. The overall malignancy rate was the same in both treatment groups. Echinocandin treated patients were also more likely to have had a solid organ transplant, diabetes mellitus, and hyperbilirubinemia.

**Table 1:** Patient characteristics by initial therapy

**Characteristic**	**Echinocandin**	**Fluconazole**	**P**
	**(n = 315)**	**(n = 374)**	
Male	168 (53%)	198 (53%)	.94
Age	58 ± 17	56 ± 21	.17
Immunosuppressive therapy within 30 days	189 (60%)	155 (41%)	<.001
Corticosteroids	184 (58%)	153 (41%)	<.001
Nonsteroid immunosuppressive therapy	43 (14%)	25 (7%)	.003
Renal injury (serum creatinine > 2 mg/dL or dialysis)	146 (46%)	124 (33%)	<.001
Dialysis (chronic or acute)	101 (32%)	77 (21%)	<.001
Any malignancy	49 (16%)	61 (16%)	.83
Hematologic malignancy	17 (5%)	8 (2%)	.025
Solid tumor	34 (11%)	55 (15%)	.14
Neutropenia within in the preceding 30 days	20 (6%)	2 (1%)	<.001
Diabetes mellitus	139 (44%)	134 (36%)	.029
Abnormal hepatic function	123 (39%)	120 (32%)	.066
Transaminitis	84 (27%)	81 (22%)	.13
Hyperbilirubinemia	87 (28%)	79 (21%)	.050
Solid organ transplant	31 (10%)	16 (4%)	.004
HIV seropositive	6 (2%)	5 (1%)	.56
Surgical patient	119 (38%)	164 (44%)	.12

Values are n (%) or mean ± SD.

### Antifungal management and *Candida* species

Use within the preceding month of an antifungal agent as prophylaxis (or possibly for a suspected but ultimately unconfirmed infection) was common, and higher among patients initially prescribed an echinocandin for their index infection than among those prescribed fluconazole [164 (52%) versus 143 (38%), P < .001] (Table 2). Patients previously given an echinocandin were unlikely to receive fluconazole for the index infection. The likelihood of receiving empiric treatment did not differ with choice of therapy, occurring in 47 (15%) of the echinocandin patients and 52 (14%) of the fluconazole patients (P = .74). Echinocandin patients were on average entered into the PATH registry three months later than fluconazole patients (P < .001).

**Table 2:** Patient characteristics by initial therapy

**Characteristic**	**Echinocandin**	**Fluconazole**	**P**
	**(n = 315)**	**(n = 374)**	
Antifungal exposure in preceding 30 days	164 (52%)	143 (38%)	<.001
Triazole exposure	92 (29%)	127 (34%)	.19
Echinocandin exposure	90 (29%)	15 (4%)	<.001
Empiric therapy	47 (15%)	52 (14%)	.74
Delay between culture and treatment, days	2.5 ± 1.4	2.2 ± 1.4	.033
Months since PATH registry opened	26 ± 11	23 ± 12	<.001
Initial therapy changed by day 2	35 (11%)	90 (24%)	<.001
Initial therapy changed by day 6	98 (31%)	121 (32%)	.74

Values are n (%) or mean ± SD.

In 334 (48%) cases *Candida albicans* was the sole cause of candidemia. *Candida glabrata* was the only species isolated in 175 (25%) cases, *parapsilosis* in 85 (12%), *tropicalis* in 51 (7%), and *krusei* in 10 (1%). Thirty-four (5%) patients had either another or multiple species isolated. Of the patients with *glabrata*, 113 (65%) had received antifungal therapy either as prophylaxis or as empiric therapy, compared to 133 (40%) of *albicans* infected patients and no more than 45% of patients infected with any other species (P < .001). Choice of initial therapy varied with species (P < .001), with 210 (63%) of *albicans* infected patients initially prescribed fluconazole and 110 (63%) of *glabrata* infected patients initially prescribed echinocandins (Table 3).

**Table 3:** Initial therapy by species

**Species**	**Echinocandin**	**Fluconazole**
	**(n = 315)**	**(n = 374)**
*Albicans*	124 (37%)	210 (63%)
*Glabrata*	110 (63%)	65 (37%)
*Krusei*	6 (60%)	4 (40%)
*Parapsilosis*	31 (37%)	54 (64%)
*Tropicalis*	24 (47%)	27 (53%)
Other species or mixed infection	20 (59%)	14 (41%)

Values are n (%).

**Figure 2:**
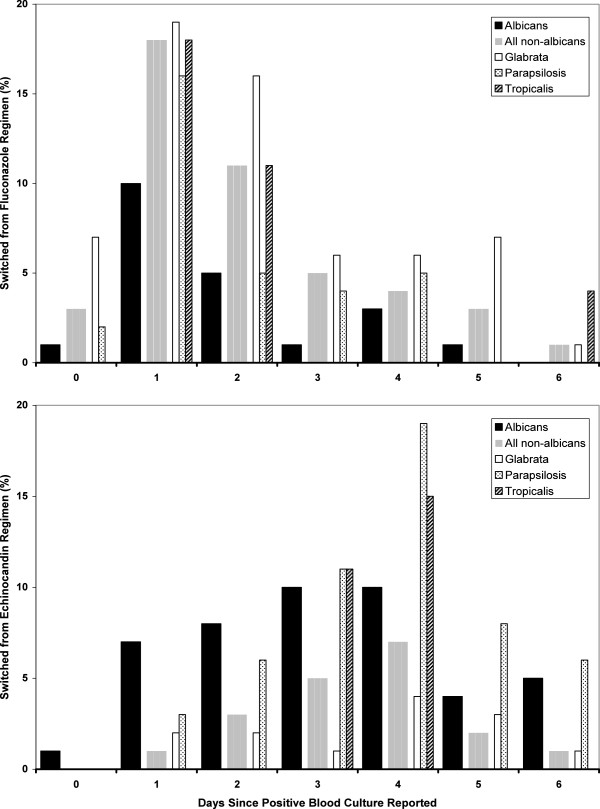
**Frequency of changes from initial therapy by day since candidemia confirmation, by initial regimen (fluconazole in upper graph, echinocandin in lower graph) and mycology.** Thick bars show patterns for *albicans* and aggregated non-*albicans* cases, thin bars individual non-*albicans* species (for species with at least 20 cases). Heights of bars represent the percent of patients within each category (treatment and species) switched on a given day.

The initial antifungal was changed or supplemented within two days more often among fluconazole than echinocandin patients [90 (24%) and 35 (11%) patients respectively, P < .001], but within six days of starting therapy changes or supplementation rates were almost identical. Changes to initial fluconazole treatment occurred most commonly one day after identification of candidemia, and on that day almost equally among patients infected with any species other than *albicans*, suggesting that early results of testing for *albicans* infection motivated the change (Figure 2). In contrast, changes to initial echinocandin regimens typically were made three to four days after identification of candidemia.

The propensity model for using an echinocandin as initial therapy included nine parameters. Increasing age, immunosuppressive therapy, dialysis, neutropenia, and previous echinocandin use all predicted a higher likelihood of echinocandin use, while empiric treatment, previous corticosteroid use, and having a non-hematologic malignancy predicted a lower likelihood. Echinocandin use also rose over time. The model’s discrimination was C = .75.

### Initial therapy and survival

**Figure 3:**
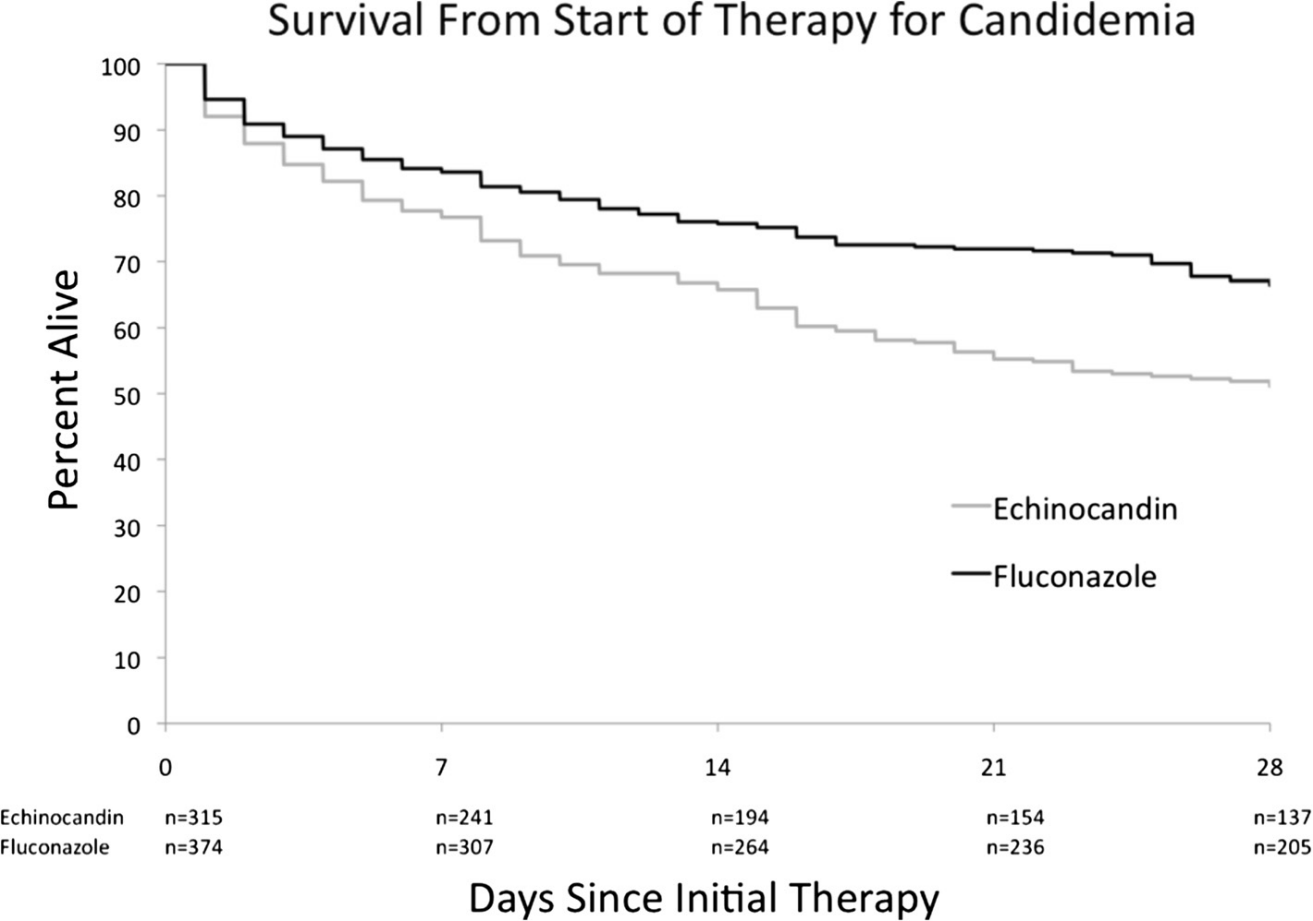
Survival through 28 days by initial therapy for candidemia.

Survival through 28 days was lower among patients initially treated with echinocandins than among those treated with fluconazole (51% and 66% respectively, P < .001) (Figure 3). Univariate proportional hazards analysis yielded a hazard ratio of .61 (.48–.78) associated with fluconazole as initial therapy.

Initial therapy with fluconazole remained a predictor of better outcome irrespective of whether therapy was changed early (within two days of starting therapy) or continued for longer periods. Among the 35 of 315 patients initially treated with an echinocandin who had antifungal therapy changed 28 day survival was 51%, no different than that of patients initially treated with an echinocandin whose therapy was not changed within two days. In contrast, among the 90 patients initially treated with fluconazole whose therapy was changed within the first two days, 28 day survival was 61%. Survival to 28 days among patients who received fluconazole alone for more than two days was 68%. Proportional hazards modeling found no significant interaction between initial therapy choice, whether or not therapy was changed within two days, and survival (P = .79 for interaction). Initial therapy with fluconazole remained a predictor of better outcome, irrespective of whether therapy was changed early.

**Figure 4:**
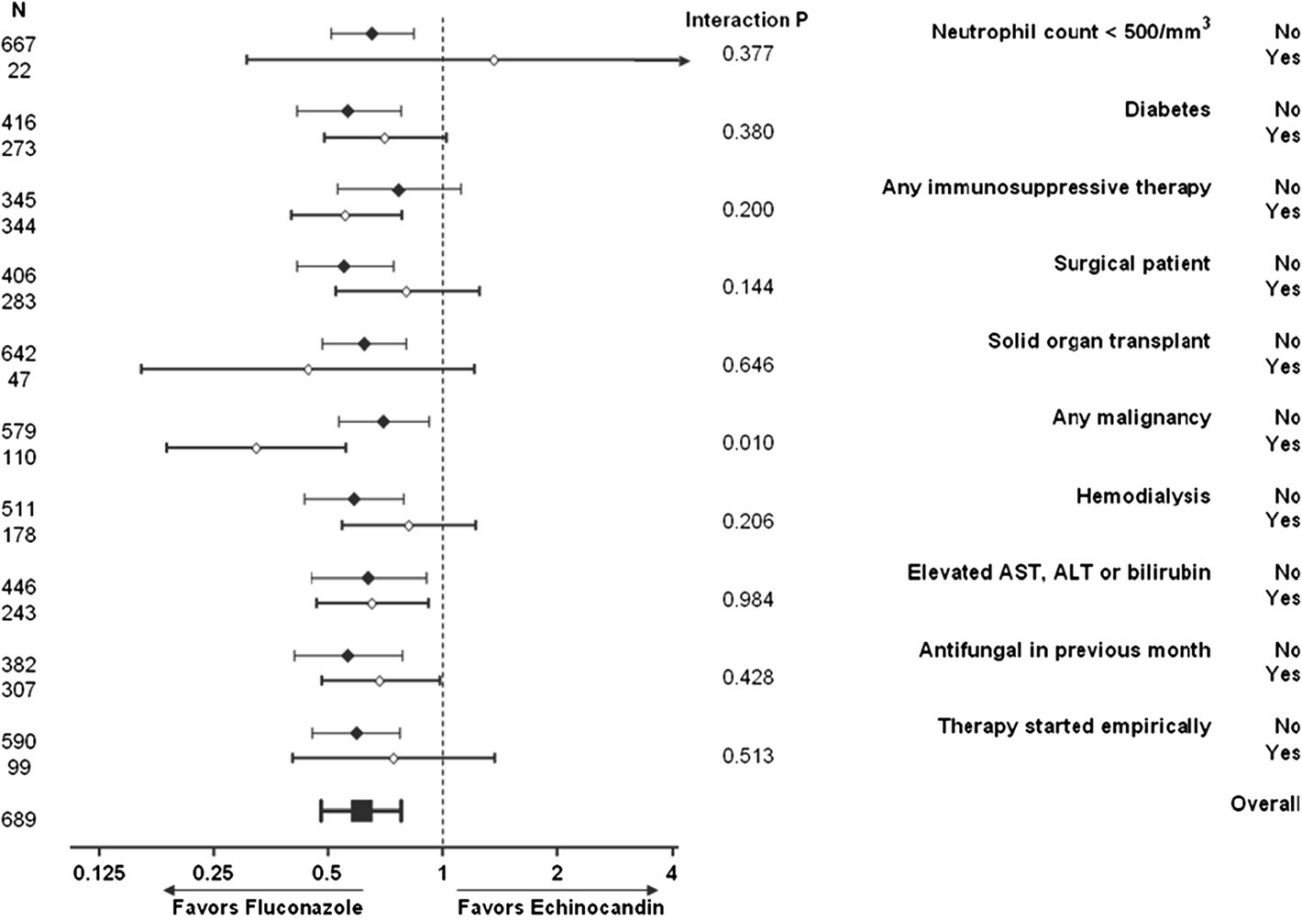
Summary analysis of treatment outcomes associated with the absence or presence of specific candidemia risk factors.

The point estimate for the hazard ratio associated with use of fluconazole as initial therapy was 1.37 (.31–6.13) among the 22 patients who had been neutropenic. For all other risk factors, point estimates of the hazard ratio associated with fluconazole use were less than one in both subpopulations with and without each risk factor (Figure 4). An interaction (P = .010) was noted between malignancy and fluconazole therapy: hazard ratio .70 (.54–.92) among the patients without malignancy, and .32 (.19–.56) among the 110 with malignancy.

**Figure 5:**
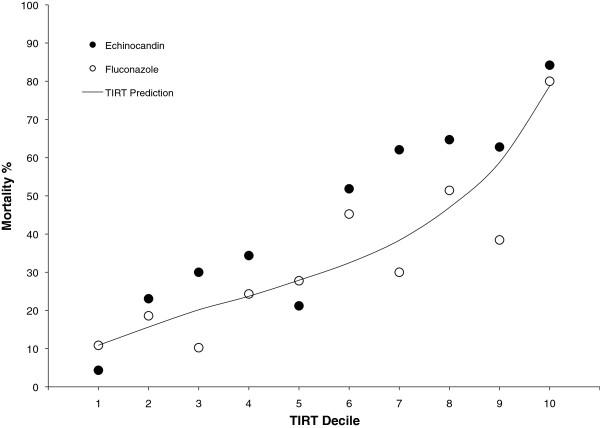
**TIRT performance over deciles of risk.** Patients were grouped into deciles by TIRT-predicted risk for death by day 28. Actual mortality for each treatment group is displayed for each decile of risk. The line demonstrates the TIRT-predicted risk for the entire decile.

The TIRT included 11 parameters. Increasing age, immunosuppressive therapy, liver disease, elevated bilirubin, dialysis, neutropenia, non-hematologic malignancy, and male gender were associated with increased risk of death, while delay between culture and treatment initiation, surgical status, and having a solid organ transplant were associated with reduced risk. Propensity for echinocandin use did not enter the model. TIRT predicted mortality rates ranged from 10% in the lowest decile of risk to nearly 80% in the highest decile (Figure 5), with C statistic of 0.74. The TIRT anticipated a 6% absolute difference in 28 day survival favoring the group treated initially with fluconazole.

In the entire study population, initial therapy was independently associated with risk of death when accounting for other risks using the TIRT in a bivariate proportional hazards model: fluconazole had a hazard ratio of .75 (.59–.96). The coefficient for TIRT, .969 (.809–1.129), remained close to unity.

The association between initial therapy choice for candidemia and survival changed little depending upon whether patients received prior or empiric antifungal therapy. Adjusting for other risk factors using the TIRT, the hazard ratio associated with fluconazole was .73 (.52–1.02) among patients who had not previously received antifungals, and .79 (.55–1.13) among those who had received prior (including empiric) therapy, .78 (.50–1.21) among those documented as having received prior but no empiric therapy, and .84 (.45–1.57) among those who received empiric therapy.

**Figure 6:**
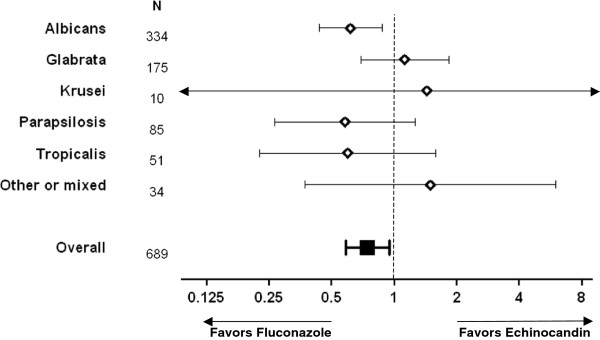
**Outcome of initial therapy by *Candida *** **species.**

Initial fluconazole therapy remained a significant predictor of survival when accounting for other risks using the TIRT among patients with *albicans* candidemia, with hazard ratio .62 (.44–.88). For other species the hazard ratios associated with initial therapy did not differ significantly from unity, but point estimates suggested increased risk of death with fluconazole therapy among patients with *glabrata* and *krusei* infection, and decreased risk among patients with *parapsilosis* and *tropicalis* infection (Figure 6).

### Discussion

Candidemia is life-threatening in critically ill patients, with a crude 30-day mortality of approximately 50% [4,23-25]. In our cohort, initial therapy with fluconazole was associated with better survival than therapy with echinocandins. Rapid initiation of antifungal therapy improves survival, but previous studies have not addressed initial treatment [10-12,26]. PATH records actual clinical practice, includes a greater number of critically ill patients than other candidemia studies, and does not exclude patients for recent antifungal therapy, prophylaxis, or comorbidity. This registry thus may provide a truer picture of the treated population than do many controlled trials. Our findings may impact treatment decisions for these patients, particularly as they are inconsistent with recently promulgated recommendations for therapy [14,27].

Uniquely, our analysis focuses on initial therapy, considered key for other severe infections. For example, inadequate initial antimicrobial therapy for bloodstream infections and pneumonia is strongly associated with increased hospital mortality [7,9]. Controlled trials of candidemia treatment have allowed as much as 48 hours of non-study antifungal therapy [16,18,19]. Re-analysis of these trials cannot address this crucial early treatment period [28].

In clinical practice initial antifungal therapy cannot usually be directed by species. Germ tube analysis, a rapid test widely available during PATH registration, only determines whether a *Candida* is *albicans*. That day 1 was the most frequent day for switching from initial fluconazole therapy suggests changes were often made when it was determined that the pathogen was not *albicans*, since response to antifungal therapy could not have been reasonably assessed by that time. Consistent with this rationale, patients infected with *parapsilosis* were as likely to be switched from fluconazole as those with *glabrata* in the first days of therapy. Newer tests, such as peptide nucleic acid-fluorescence in situ hybridization (PNA-FISH), have accelerated speciation in some centers, but the majority of patients with candidemia are started on therapy before the species is determined [29].

Initial therapy often differs from later tailored therapy, particularly if resistance is present. Some forms of resistance may not be relevant during early therapy; for example, fluconazole resistance in *Candida glabrata* is mediated by inducible enzymes that may not be expressed early in the course of treatment [30]. Intrinsic *Candida* resistance to fluconazole is relatively rare, found in 9–15% of *glabrata*[9] (25% of registry cases), and in *krusei* (fewer than 2% of cases), therefore impacting fewer than 5% of patients with candidemia overall [13]. Resistance to both echinocandins and fluconazole has been reported from a large international survey of clinical *Candida* isolates [31].

Initial therapy for suspected or proven candidemia in the ICU may be chosen based upon severity of illness, sensitivities of probable infecting species, physician habit and other factors. Published guidelines, based largely on expert opinion, have expressed a preference for an echinocandin over fluconazole as initial therapy for moderately to severely ill patients, a criterion satisfied by all of the patients included in our study [4,14]. The apparent inferiority of echinocandins as initial therapy in the PATH population stands in contrast to this recommendation; if the guideline is correct, the impact of unmeasured variables in our study would have to be sufficient to not only account for the observed survival difference but to reverse it.

Whether initial antifungal therapy was changed in the first days of treatment or not did not influence the association of therapy choice and survival. If fluconazole therapy were indeed inferior to echinocandin therapy, one would expect patients started and continued on echinocandins to have been more likely to survive than patients started on fluconazole and then only later switched to another agent (whether because of clinical failure or laboratory findings). This was not the case however: patients started on fluconazole and then switched to another antifungal regimen within two days were more likely to survive than those initially treated with an echinocandin.

While the propensity score accounts for some of the differences that might have led to use of an echinocandin instead of fluconazole, it almost certainly does not encompass all factors contributing to prescribing decisions. Beyond this, we believe uniformity of practice is unlikely over the diversity of sites and prescribers. Subset analyses also demonstrate that the PATH registry’s possible conflation of empiric and previous prophylactic antifungal use cannot account for the differences in survival.

It is unlikely that differences in underlying disease account for the survival differences we observed between treatment groups. Studies of the critically ill have consistently found that, once ongoing physiology is accounted for, underlying diseases impact short-term mortality only when present in their most severe forms [32-34]. Data regarding such conditions were captured by PATH and included in regression models.

The PATH registry captures data on several organ dysfunctions associated with mortality in critically ill septic patients and typically incorporated into acuity models: respiratory (present in all patients included in this analysis), renal, and hepatic. These data were included in the development process for the TIRT and propensity models. The performance of the TIRT was comparable to that of other composite risk models, such as APACHE II, when used to stratify patients with severe infections [35,36].

An obvious potential confounder not recorded in the PATH database was presence or absence of shock. The addition of vital signs data to those incorporated in the TIRT is unlikely to appreciably improve stratification of these patients [36]. All patients studied satisfied severe sepsis criteria, with candidemia and at least one organ failure. Cardiovascular dysfunction in this setting is generally associated with a further attributable mortality risk of approximately 5–7% [37]. This could account for some of the difference in outcome we observed if almost all patients with hypotension were treated initially with echinocandins, and almost all patients treated with echinocandins were hypotensive when therapy was started. This is highly improbable, since prescribing habits are seldom so uniform across centers, particular in the absence of compelling pre-existing data. A recent large cross-sectional study found virtually identical vasopressor use and severity of illness scores among candidemia patients treated with fluconazole or an echinocandin [38].

Our results differ from the prospective, randomized trial in patients with candidemia and invasive candidiasis comparing fluconazole and anidulafungin, which demonstrated improved clinical and microbiological response in the echinocandin arm [19]. In a retrospective analysis of the moderately severe to severely ill patient subset from this trial the trend toward improved global response persisted [39]. A recent patient-level review of candidemia trials reported improved clinical outcomes in patients receiving an echinocandin. In subgroup analysis, this benefit was restricted to the less sick half of the analyzed population [28]. The validity of this published analysis has also been questioned, as restriction to the more current studies eliminates the reported benefit of echinocandin therapy [40]. Because of study design, the effect of initial therapy cannot be addressed.

## Conclusions

Our study represents the largest investigation of initial antifungal therapy for candidemia in mechanically ventilated patients. The results favor fluconazole, contrasting with several recent recommendations and guidelines reflecting expert opinion but only limited clinical data. Despite the challenges, we believe a large, controlled clinical trial comparing fluconazole and an echinocandin as initial antifungal therapy for candidemia in the critically ill is warranted.

## Key messages

– Randomized controlled trials of therapy for candidemia in the critically ill have not evaluated initial therapy, considered critically important in the treatment of other types of infections.

– In a large, multicenter registry of fungal infections, mortality was lower among mechanically ventilated patients initially treated with fluconazole than among those treated with an echinocandin. These mortality differences persisted after adjusting for severity of acute and chronic illness.

– Currently published guidelines lean toward the use of echinocandins in the treatment of candidemia in critically ill patients. A large, randomized trial is justified to identify the optimal initial management of this population.

## Abbreviations

PATH: Prospective antifungal therapy; TIRT: Treatment independent risk term; PNA-FISH: Peptide nucleic acid-fluorescence *in situ* hybridization.

## Competing interests

MF and AQ have no conflicts of interest. DK has been a consultant for Pfizer and Astellas and a speaker for Astellas, Glaxo, and Pfizer. MM has been a consultant for Astellas, Merck, and Pfizer, and a speaker for Astellas and Pfizer. MM has received research funding from Astellas, Basilea, and Pfizer.

## Authors’ contributions

Each of the authors participated in the study design, data analysis, manuscript preparation and editing, and preparation of tables and figures. AQ performed the primary data analyses. MM was a site principal investigator in the PATH database and actively participated in data collection. All authors read and approved the final manuscript.

## Authors’ information

Dr. Ferrada is currently enrolled in a combined Critical Care Medicine & Infectious Diseases fellowship training program at the National Institutes of Health and Johns Hopkins School of Medicine.

## Pre-publication history

The pre-publication history for this paper can be accessed here:

http://www.biomedcentral.com/1471-2253/13/37/prepub
